# The Ca^2+^ permeation mechanism of the ryanodine receptor revealed by a multi-site ion model

**DOI:** 10.1038/s41467-020-14573-w

**Published:** 2020-02-17

**Authors:** Aihua Zhang, Hua Yu, Chunhong Liu, Chen Song

**Affiliations:** 1 Center for Quantitative Biology, Academy for Advanced Interdisciplinary Studies, Peking University, Beijing, 100871 China; 2 Peking-Tsinghua Center for Life Sciences, Academy for Advanced Interdisciplinary Studies, Peking University, Beijing, 100871 China

**Keywords:** Computational biophysics, Permeation and transport

## Abstract

Ryanodine receptors (RyR) are ion channels responsible for the release of Ca^2+^ from the sarco/endoplasmic reticulum and play a crucial role in the precise control of Ca^2+^ concentration in the cytosol. The detailed permeation mechanism of Ca^2+^ through RyR is still elusive. By using molecular dynamics simulations with a specially designed Ca^2+^ model, we show that multiple Ca^2+^ ions accumulate in the upper selectivity filter of RyR1, but only one Ca^2+^ can occupy and translocate in the narrow pore at a time, assisted by electrostatic repulsion from the Ca^2+^ within the upper selectivity filter. The Ca^2+^ is nearly fully hydrated with the first solvation shell intact during the whole permeation process. These results suggest a remote knock-on permeation mechanism and one-at-a-time occupation pattern for the hydrated Ca^2+^ within the narrow pore, uncovering the basis underlying the high permeability and low selectivity of the RyR channels.

## Abstract (web summary)

Although the permeation mechanisms for K^+^ and Na^+^ channels have been extensively studied, the ion permeation mechanism through Ca^2+^ channels was largely unknown. Here the authors develop a multisite Ca^2+^ model that can be used in the framework of classical MD simulations to study Ca^2+^ in a quantitative manner, and use it to investigate the ion permeation mechanism of the ryanodine receptor 1.

## Introduction

As an essential messenger in cells, calcium ions (Ca^2+^) regulate many physiological processes, including neurotransmitter release, muscle contraction, and hormones secretion^1^. The concentration of Ca^2+^ in the cytoplasm and organelles is precisely controlled by multiple calcium channels, including the voltage-gated calcium channels in cell membranes and the ryanodine receptors (RyR) in the endoplasmic reticulum (ER) membrane. Ca^2+^ also induces conformational changes of a wide range of Ca^2+^-interacting proteins, such as calmodulin and Ca^2+^-activated ion channels, to trigger downstream signal transduction^2^. Although the pathways of calcium signaling are extensively studied, the molecular interaction details between calcium and proteins have yet to be fully elucidated.

To study the detailed interactions between ions and proteins, we can often use molecular dynamics (MD) simulations to provide microscopic and quantitative insights, thereby obtaining the specific functional mechanisms of the relevant proteins^3–6^. However, the conventional models of Ca^2+^ are far from accurate in calculating the interaction energies between Ca^2+^ and proteins^7–9^, therefore inadequate to study the precise Ca^2+^-protein interactions. As a consequence, K^+^ and Na^+^ channels have been widely studied by MD simulations, and their detailed permeation mechanisms were revealed^10–17^, but computational studies of Ca^2+^ channels are rather limited. Notably, several structures of Ca^2+^ channels were resolved recently^18–21^, which provided a solid basis to study their detailed function mechanism further. In particular, the open-state RyR1 channel provides an excellent opportunity for studying Ca^2+^ permeation and selectivity^21^, which makes a reliable Ca^2+^ model even more desirable.

**Fig. 1:**
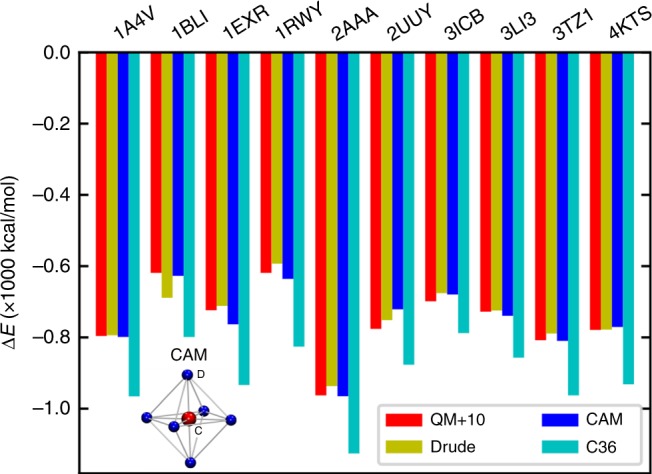
Calcium-protein binding energies calculated with different methods. The labels on the *x*-axis represent the PDB IDs of the ten Ca^2+^-bound proteins for which we performed the binding energy calculations. The legend indicates the methods of the calculation, QM+10: corrected quantum mechanics calculation; CAM: our multi-site Ca^2+^ model; Drude: the polarizable Drude force field; C36: the CHARMM36 force field. Our multi-site Ca^2+^ model consists of a central atom (C) and six dummy atoms (D) located at the vertices of an octahedron, as shown with the inset. Source data are provided as a Source Data file.

There have been enormous efforts in trying to develop a more accurate Ca^2+^ model. The polarizable force field is theoretically appealing^9^, but its implementation and validation still need further work before being widely accepted and utilized for membrane protein simulations. Kohagen et al. proposed to scale the partial charges on the Ca^2+^ to account for the charge transfer and polarization^22,23^. Another strategy is to represent an ion by distributing electrostatic and Lennard-Jones (LJ) interactions on multiple sites, which can introduce a much larger parameter space and therefore make the ion model more tailorable^24–26^. Unfortunately, none of the existing Ca^2+^ models in the non-polarizable classical force field are able to describe the interactions between Ca^2+^ and protein quantitatively. Therefore, in the present work, we developed a new multi-site Ca^2+^ model particularly optimized for Ca^2+^-protein interaction (inset of Fig. 1), and then utilize this model to study the detailed Ca^2+^ permeation mechanism through the RyR1 channel, which showed distinct features from the widely studied K^+^ and Na^+^ channels.

## Results

### The multi-site Ca^2+^ model behaves well in water and protein

We designed a seven-site ion model, as shown in the inset of Fig. 1. There are six adjustable parameters, including *b*_CD_, *Q*_C_, *ε*_C_, *σ*_C_, *ε*_D_, and *σ*_D_, where *b*_CD_ is the distance between dummy atoms and the central atom, *Q*_C_ is the charge on the central atom, and *ε* and *σ* are the LJ parameters of the central (C) and dummy (D) atoms, respectively. We further distinguish the LJ interactions of Ca^2+^ with water and non-water by replacing (*ε*_C_, *σ*_C_) with two sets of ($\varepsilon _{\mathrm{C}}^{\mathrm{W}}$, $\sigma _{\mathrm{C}}^{\mathrm{W}}$) and ($\varepsilon _{\mathrm{C}}^{{\mathrm{NW}}}$, $\sigma _{\mathrm{C}}^{{\mathrm{NW}}}$), respectively. The charges on all the dummy atoms are the same and determined so that the total charge of the model is +2*e*. By adjusting the aforementioned parameters (Supplementary Fig. 1), we obtained a Ca^2+^ model that can quantitatively reproduce the energetical and dynamic properties of Ca^2+^ in water as well as the Ca^2+^-protein interactions (please refer to the method section and Supplementary Tables 1 and 2 for details). The resulting Ca^2+^ properties in water are shown in Table 1. As can be seen, the hydration free energy (Δ*G*_h_), the first-peak position of the radial distribution function of water around Ca^2+^ (*R*_1_), and the number of coordinated water molecules in the first solvent shell (*N*_C_) have all reached the target values of experiments. In addition, the residence time of water molecules in the first solvation shell (*τ*_R_) can be optimized to below 100 ps, which was consistent with the previous experimental estimation^27,28^, and solved the common problem of Ca^2+^ being too sticky. We also performed ab initio MD simulations, and observed multiple exchange of water molecules between the first and second solvation shells within 200 ps, providing additional support that the residence time of our model is reasonable (Supplementary Fig. 2). During the model optimization procedure, it was interesting to see that the water residence time in the first solvation shell can be optimized to a considerable extent with the multi-site model, as models with different parameters can show very close hydration energies while residence time differing by two orders of magnitude.

**Table 1:** The target properties of Ca^2+^ in water and the computed property values from the simulations with our Ca^2+^ model.

	Δ*G*_h_ (kJ/mol)	*R*_1_ (nm)	*N*_C_	*τ*_R_ (ps)
Target	−1504.0	0.242	7.0	~100
Computed	−1503.9	0.242	7.0	75

With our model, the calculated binding energies of Ca^2+^ and proteins were also improved to a large extent (Fig. 1). The default Ca^2+^ parameters of the CHARMM force field (C36) led to a significant overestimation of 150–200 kcal/mol^9^, while the average binding-energy discrepancies for ten selected proteins was −0.2 kcal/mol for our multi-site model. Therefore, our model is comparable to the quantum mechanics (QM) and polarizable Drude model in calculating the Ca^2+^-protein binding energies (Fig. 1 and Supplementary Table 1). While in the meantime, our multi-site Ca^2+^ model is compatible with the classical non-polarizable force field, allowing immediate and efficient simulations of complex Ca^2+^ interacting proteins.

We then further validated the thermodynamic property of our Ca^2+^ model around a protein by calculating the potential of mean force (PMF) of pulling a Ca^2+^ away from its binding site in a calpain. As can be seen in Supplementary Fig. 3, our model predicted a **Δ***G*_PMF_ of ~33 kJ/mol and binding free energy of ~25 kJ/mol, while the default Ca^2+^ parameters of the CHARMM force field resulted in a much larger **Δ***G*_PMF_ of ~177 kJ/mol and binding free energy of ~168 kJ/mol. The experimental binding affinity is ~35 kJ/mol^29^, indicating that our Ca^2+^ model is more accurate in reproducing not only the potential energies but also the free energies in the study of Ca^2+^-protein interactions.

### The permeability of the open-state RyR1

**Fig. 2:**
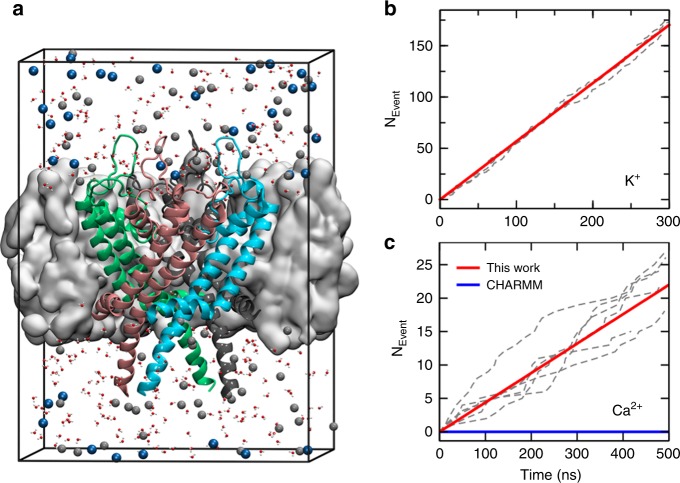
Simulation of ion permeation. **a** The simulation system of Ca^2+^ (gray spheres) permeation through the RyR1 channel. The pore domain of RyR1 (cartoon) is embedded in a POPC membrane (gray surface). Cl^−^ ions are shown with blue spheres. Only part of water molecules (small red and white spheres) are shown for clarity. **b** The cumulative number of K^+^ ions permeating through the RyR1 channel as a function of simulation time. The dashed gray lines correspond to three independent simulation trajectories, and the solid red line corresponds to the average of the total permeation count. The conductance calculated from these trajectories is 910 ± 39 pS. **c** Same as **b** but for Ca^2+^ ions. The conductance from six trajectories is 141 ± 30 pS with our Ca^2+^ model. Source data of **b** and **c** are provided as a Source Data file.

The conventional ion models generally work well in studying ion-protein interactions for K^+^ or Na^+^, but fail consistently for Ca^2+^ ions^9^, and therefore no calcium permeation was observed in previous MD studies of Ca^2+^ channels^30^. We performed MD simulations on the type-1 ryanodine receptor (RyR1), an intracellular calcium release channel required for skeletal muscle contraction, with our Ca^2+^ model. The open-state structure of RyR1 (PDB ID: 5TAL [10.2210/pdb5TAL/pdb]) was obtained from des Georges et al.’s^21^ work. The simulation systems consist of the pore domain of RyR1 embedded in a lipid bilayer of 1-Palmitoyl-2-oleoyl-sn-glycerol-3-phosphocholine (POPC) and an aqueous solution of either 150 mM Ca^2+^ or 250 mM K^+^ (Fig. 2a). The protein was restrained to the open-state crystal structure, and a transmembrane potential of 100 mV was applied along the direction from the sarcoplasmic reticulum (SR) lumen to the cytosol.

We first studied the permeation of K^+^ through RyR1 as a test as well as a validation. Three independent 300-ns MD simulations were conducted and generated enough permeation events (~500) for statistical analysis (Fig. 2b). The conductance was calculated to be 910 ± 39 pS, which was close to the experimentally measured saturated K^+^ conductance of ~1 nS and indicates that the RyR1 structure under study is indeed in its open state and that the K^+^ model in the CHARMM force field is reasonably accurate in describing the interactions between ions and proteins. However, our MD simulations of Ca^2+^ permeation through the same open-state RyR1 showed that the channel is not permeable to Ca^2+^ at all with the default Ca^2+^ parameter of CHARMM. Not a single permeation event was observed in three 500-ns MD trajectories (Fig. 2c). Since the channel is in its open state, this indicated that the default Ca^2+^ model gave us qualitatively wrong simulation results here, as also observed by another recent study^30^. A close inspection of the MD trajectories showed that the Ca^2+^ ions were tightly bound to the protein, again confirming that the binding affinity between Ca^2+^ and protein is too strong with the conventional Ca^2+^ model. In contrast, when our Ca^2+^ model was used for the same simulations, we observed continuous Ca^2+^ permeation as expected (Fig. 2c). The conductance calculated from six 500-ns trajectories is 141 ± 30 pS, which agrees reasonably well with the experimental value of ~172 pS with the same ion concentration^31^. Therefore, we believe that our multi-site Ca^2+^ model is indeed more accurate in studying the permeation behavior of the Ca^2+^ channel.

### The Ca^2+^ binding sites in the pore region

**Fig. 3:**
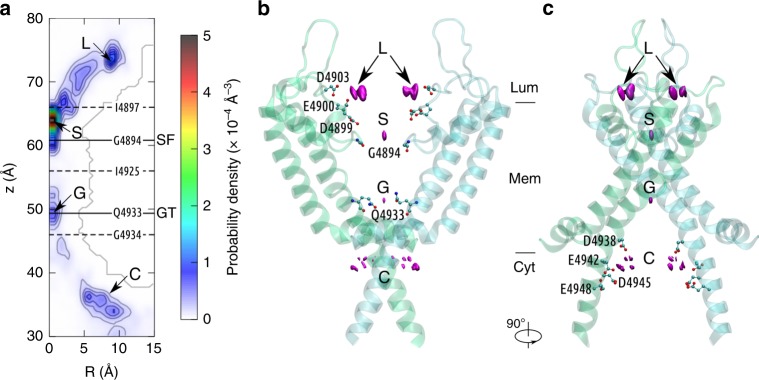
The Ca^2+^ binding sites in the RyR1 channel. **a** The contour plot of Ca^2+^ density on the R-z plane around RyR1. Four binding sites within the pore are designated as L, S, G, and C. The positions of the selectivity filter (SF) and gate (GT) constrictions are indicated with solid lines. The ion channel is divided into chambers by the dashed lines at the density saddles. The pore residues in close proximity are labeled on these lines. **b**, **c** Side views of the RyR1 channel. Only two chains are shown for clarity. The bottleneck residues (GLY-4894 at SF and GLN-4933 at GT), and the negatively charged residues at the binding sites L and C are shown as ball-and-sticks. Source data of (**a**) are provided as a Source Data file.

We performed an 800-ns simulation without the transmembrane potential to identify the Ca^2+^ binding sites in RyR1. The contour plot of the Ca^2+^ density, *ρ*(*R*, *z*), is presented in Fig. 3a, from which two major binding sites in the luminal vestibule (L) and above the selectivity filter constriction (S), and one minor binding site at the gate constriction (G) can be identified within the transmembrane pore. The selectivity filter and gate constrictions are located near the residues G4894 and Q4933, respectively (Fig. 3b). The corresponding positions in the contour plot are indicated by solid lines labeled with SF and GT in Fig. 3a. The isosurfaces of probability density corresponding to these binding sites are shown in Fig. 3b. By calculating the residence time of carboxylate oxygen of negatively charged residues within a sphere with a radius of 5 Å around the binding site L, we identified that the binding site L is formed by the interaction of Ca^2+^ ions with D4899, E4900, and D4903 residues (Fig. 3b), which agrees well with previous experimental and computational studies^30,32^. The large probability of finding Ca^2+^ in the selectivity filter also indicates that the Ca^2+^ can easily accumulate around the luminal vestibule and move into the upper filter, and the rate-limiting step of permeation is the process of passing through the lower selectivity filter or gate constrictions. In addition, a continuous cytosolic binding region (C) was found near residues D4938, E4942, and D4945, which interact with the permeating Ca^2+^ and may influence the ion permeability as well. This is consistent with a previous experimental study showing that D4938 and D4945 determine the ion flux and selectivity^33^.

### The Ca^2+^ ions are fully hydrated during permeation

**Fig. 4:**
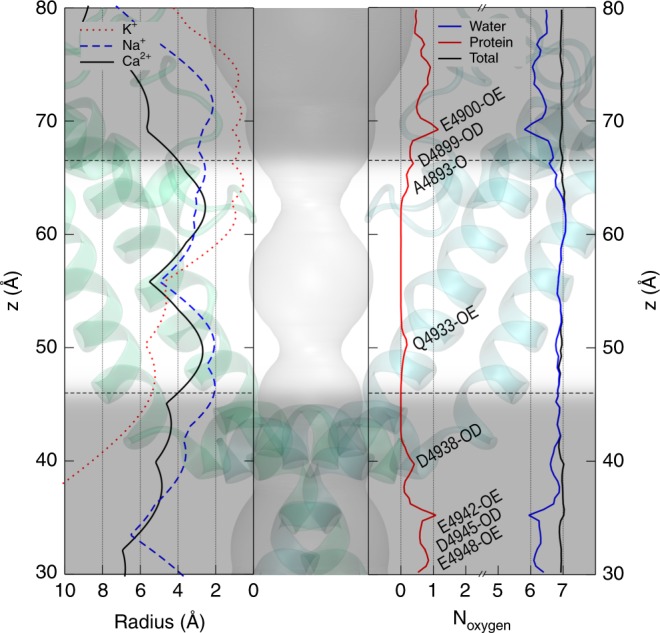
Ca^2+^ ions are fully hydrated during permeation. The left panel shows the pore radii along the pore axis of the open-state RyR1 (black solid), K^+^ channel (red dotted), and Na^+^ channel (blue dashed). The right panel shows the total number (black) of coordinated oxygen atoms around the calcium ions within the pore, and the contributions from protein (red) and water (blue) respectively. The significant contributions from protein oxygen are marked with corresponding protein residue IDs containing the oxygen. The transparent background shows the pore profile within the open-state RyR1 structure, and the narrow pore region is highlighted between the black dashed lines. Source data are provided as a Source Data file.

We calculated the number of oxygen atoms coordinated with the permeating Ca^2+^ and monitored from which residues these oxygen atoms were (water or protein). As shown in Fig. 4, the open-state RyR pore is relatively wide compared to K^+^ and Na^+^ channels. As the first solvation shell is around 2.4 Å from the Ca^2+^ and the radius of a water molecule is usually considered to be 1.4 Å, we consider the pore region with a radius < 4.0 Å to be the narrow pore region that contains the rate-limiting constriction sites. Interestingly, the average number of oxygen atoms coordinated with the Ca^2+^ was almost constant during the permeation process of Ca^2+^, as shown in the right panel of Fig. 4, and nearly all of these oxygen atoms were from water molecules within the narrow pore region. This clearly indicates that the permeating Ca^2+^ ions do not need to dehydrate when permeating through the open-state pore, and therefore the first solvation shell was intact in the narrow pore region. On the other hand, in the wide upper selectivity filter, one of the water molecules coordinated with Ca^2+^ can be replaced by the oxygen from E4900 (Fig. 4), simply because the strong electrostatic attraction between Ca^2+^ and E4900 pushed one of the coordinated water molecules away. A similar phenomenon was observed near D4938. It should be noted that, the water replacement at these sites are not caused by the steric dehydration when ions passing through a narrow pore as observed in K^+^ and Na^+^ channels, but rather due to strong electrostatic attraction from negatively charged residues in a wide vestibule (*r* > 5 Å), and therefore should not be considered as dehydration due to the permeation.

### The Ca^2+^ permeation pattern

**Fig. 5:**
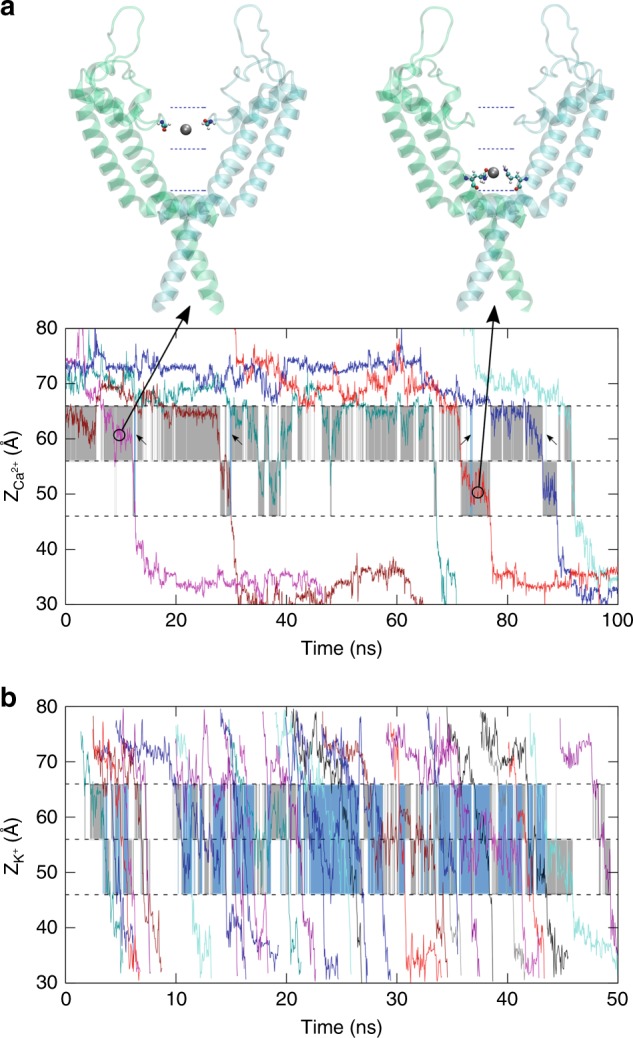
Evolution of the z coordinates when multiple ions permeate through the RyR1 channel. **a** For Ca^2+^, the occupied chamber accommodating either the selectivity filter or gate is shaded in gray unless both of them are occupied, which is indicated by blue bars depicted with small arrows. Representative configurations with one of the chambers occupied by a Ca^2+^ ion (in silver) are shown above. **b** For K^+^, similar to above, the gray shaded areas indicate one-ion occupancy while the blue shaded areas indicate two-ion occupancy. Source data are provided as a Source Data file.

The narrow region of the channel consists of the lower selectivity filter, the cavity, and the gate, which can be divided into two chambers by the saddle points of the Ca^2+^ density, as indicated by the dashed lines in Fig. 3a. The upper and lower chambers contain the binding sites S and G, respectively. The typical permeation pattern of Ca^2+^ ions is shown in Fig. 5a, from which it seems that permeation through the narrow pore region of the channel occur mainly in a one-at-a-time manner, meaning that there is only one Ca^2+^ residing in this narrow pore region, either in the upper or the lower chamber. The probability of both chambers being occupied by Ca^2+^ was only 2.4% in the trajectories, while the probability of only one chamber being occupied was 68.6%. Therefore, most of the time, only one Ca^2+^ can occupy the narrow pore region when permeating through the open-state channel. However, this does not mean that other Ca^2+^ are not involved in the permeation process. In fact, we can see that the exit of the lower Ca^2+^ from the narrow pore is often accompanied by the (transient) entering of a second Ca^2+^ (blue bars in Fig. 5a). This indicates that, Ca^2+^ ions do not have to be in direct contract or adjacent positions to influence each other in the RyR channel. This permeation pattern is distinct from K^+^ and Na^+^ channels, in which usually both the narrow selectivity filter and cavity can be occupied by multiple permeating ions at the same time^11,13,16^. This is probably due to the fact that the electrostatic repulsion is much stronger between divalent ions than monovalent ions (Supplementary Fig. 4), and that RyR1 has a much shorter narrow selectivity filter region than typical K^+^ and Na^+^ channels (Fig. 4). Interestingly, when K^+^ permeating through the RyR1 channel, there is a significant fraction of the time in which two K^+^ ions were found occupying the narrow pore region (Fig. 5b), which is consistent with Miranda et al.ʼs work^34^.

### Free energy of Ca^2+^ within the RyR1

**Fig. 6:**
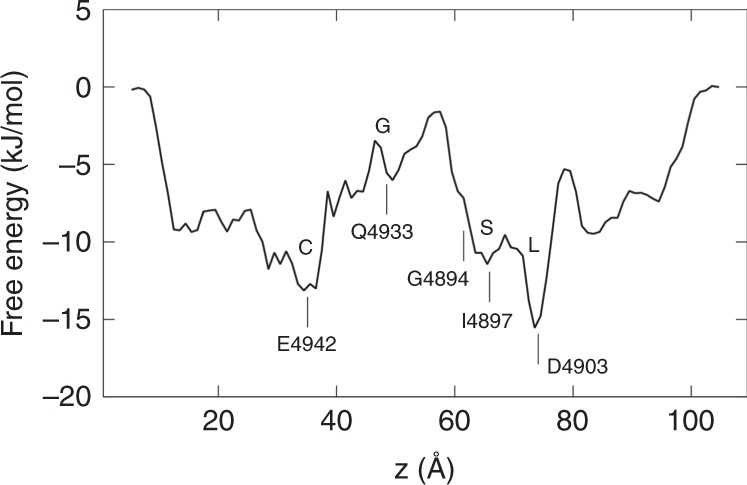
The free energy profile of Ca^2+^ along the transmembrane pore of the RyR1. The binding sites and some relevant residues are labeled. The rate-limiting free energy barrier between S and G is estimated to be ~9.8 kJ/mol. Source data are provided as a Source Data file.

We calculated the PMF of Ca^2+^ within the pore using the equilibrium MD simulation trajectories. The result is shown in Fig. 6. As can be seen, the binding sites we observed in our simulations correspond well with the free energy wells in Fig. 6, and the free energy barrier between S and G corresponds to the rate-limiting step we found in the simulation trajectories, which is only ~9.8 kJ/mol. This is consistent with our finding that Ca^2+^ ions do not dehydrate its first solvation shell when passing through this region, as the dehydration energy of Ca^2+^ is very high. We believe the energy barrier here mainly represents an electrostatic effect, due to the existence of the multiple negatively charged residues in the upper SF vestibule, as well as the minor Ca^2+^ binding site G, which can accommodate one Ca^2+^ that will hinder other Ca^2+^ from approaching the cavity by electrostatic repulsion. Such a low free energy barrier also explains why we observed a large conductance of Ca^2+^ through RyR1. The energy barrier between the binding sites G and C is even lower (~2.5 kJ/mol), indicating the gate constriction is not the rate-limiting site and Ca^2+^ will not occupy the G site for a long time before exiting the narrow pore.

## Discussion

The interaction between Ca^2+^ and protein is of great importance in studying Ca^2+^-mediated biological processes. Although the multi-site ion model cannot explicitly represent the charge transfer and polarization effect, the simulation results showed that our new model behaves much better than conventional single-point ion models. Moreover, our model is entirely consistent with the currently widely used non-polarizable force fields, such as CHARMM and AMBER, and therefore can be easily used in MD simulations. As shown in the result section, our seven-site Ca^2+^ model can reproduce the solvation properties of Ca^2+^, including the hydration energy, the first solvation shell size (first-peak position of RDF), the coordination number, and the residence time of water in the first solvation shell, as well as the Ca^2+^-protein binding energies in a more quantitative way that is comparable to quantum chemistry calculations. The optimized Ca^2+^ model was further validated by thermodynamic calculations and then utilized in the simulations of the RyR1 ion channel. In contrast to the conventional Ca^2+^ model, where ions get stuck in the channel in MD simulations, our new model resulted in the continuous permeation of Ca^2+^ through the channel, and the calculated conductance is in good agreement with the experimental electrophysiology result. Therefore, we believe this multi-site Ca^2+^ model can be widely used in simulating many Ca^2+^-involved bio-systems in addition to ion channels, and our simulations of RyR can provide detailed information about the Ca^2+^ permeation mechanism.

The ion binding and permeation mechanisms have been widely studied for K^+^ and Na^+^ channels with molecular dynamics simulations^10–12,14–17^, but only rarely studied for Ca^2+^ channels with conventional ion models^30,34^. Very recently, Heinz et al. performed extensive PMF calculations of various cations in the RyR1, and located the key binding sites within RyR1 as well as the monovalent cation permeation pathways^30^. Miranda et al. used monovalent cations and Cl^−^ to explore the permeability and origin of the high selectivity of cations over anions of RyR2^34^. Both work provided highly valuable insights on the ion permeability and selectivity of RyR channels, but the Ca^2+^ permeation details remain unclear due to the absence of a reliable Ca^2+^ model. From our MD simulations with the new multi-site Ca^2+^ model, the major Ca^2+^ binding sites in the open-state RyR1 were determined to be near the D4900 and G4894 residues on the luminal side, which is consistent with previous experiments and simulations^30,32–34^, and the rate-limiting step of permeation is found to be the step of passing through the selectivity filter constriction, where Ca^2+^ ions dwell for a relatively long time before passing through due to the existence of a free energy barrier here (Figs. 5a and 6). Our simulation indicated that Ca^2+^ can easily accumulate in the wide upper selectivity filter, as shown by the high Ca^2+^ density from our equilibrium simulations (Fig. 3a). On average, there were about five Ca^2+^ or eight K^+^ in the selectivity filter in the presence of a 100-mV transmembrane potential (Supplementary Fig. 5). This indicates that K^+^ cannot fill up the electrostatic energy well in the selectivity filter as effectively as Ca^2+^ do, implying that the selectivity among cations is mainly achieved by the charge-space competition, as hypothesized by a previous experimental study^35^. Since Miranda et al. already showed that the highly negatively charged SF vestibule is also responsible for the selectivity of cations against anions, we conclude that the highly charged rings in the SF is responsible for not only the high selectivity between cations and anions, but also the low selectivity among divalent and monovalent cations.

Na_V_ and TRP channels have a shorter SF, thus sharing a higher structural similarity with RyR channels than K_V_ channels, but their SFs are still longer than RyR1. Previous studies showed that there are multiple Na^+^ occupying the SF of Na_V_ channels, and they permeate by the so-called “loosely coupled knock-on” mechanism^13^, in which multiple partially hydrated Na^+^ interact each other within the SF and cavity. Darré et al. observed at least three monovalent or divalent cations can co-exist in the narrow pore region of TRPV1^36^. Recent structural and MD studies of the TRPV6^37,38^, a calcium-permeable ion channel, also showed that Ca^2+^ can tightly bind to the SF, and one or two additional Ca^2+^ need to approach it, line up and knock it off from the binding site. There are multiple Ca^2+^ occupying the narrow SF and cavity during this process. This is named the “knock-off” mechanism, which probably determines the ion selectivity of the Ca_V_ and TRPV6 channels^37,39,40^. We did not observe such a well-organized “knock-on” or “knock-off” phenomena in our simulations of RyR1. As shown in Fig. 3a, there is only one Ca^2+^ binding site in the narrow SF, and the other Ca^2+^ ions in the upper SF do not line up, which may still push and knock off the lower Ca^2+^ in the SF, but in a loosely coupled manner.

From our simulations, Ca^2+^ ions permeate through the narrow pore region of the RyR1 channel following a “remote knock-on” mechanism and one-at-a-time manner (Fig. 5a), which is distinct from the well-studied direct knock-on mechanism in K^+^ channel and loosely coupled knock-on in Na^+^ channels^11,13,16^. Previous studies have shown that multiple monovalent ions can enter the narrow pore region of the K^+^ and Na^+^ channels, and line up to facilitate the so-called ‘knock-on’ permeation, either tightly or loosely coupled^10–16^. This was also observed for K^+^ in RyR^34^. However, it is not the case for the Ca^2+^ in the RyR1 channel, as only one Ca^2+^ was observed in the narrow pore region (*r* < 4 Å) during the Ca^2+^ permeation in our MD simulations, and this region contains both the lower selectivity filter and cavity. There may be two reasons for this different permeation pattern, one is that the narrow selectivity filter of RyR is shorter compared to that of K^+^ and Na^+^ channels (Fig. 4), which can hardly accommodate multiple ions, and the second reason is that the electrostatic repulsion between divalent ions is much stronger than monovalent ions, which makes it energetically unfavorable for multiple divalent cations to sit side by side within a certain distance. In fact, we observed that when there was a Ca^2+^ in the cavity, other Ca^2+^ cannot stay in the lower selectivity filter (Fig. 5a). However, this does not mean other Ca^2+^ ions are not interacting with the permeating Ca^2+^. By calculating the electrostatic potential profile (Supplementary Fig. 4), we can see that the Ca^2+^ in the lower binding site can exert a large force on those Ca^2+^ in the SF, and vice versa. Therefore, even though there is only one Ca^2+^ ion in the narrow pore region, those Ca^2+^ in the SF can still electrostatically push it. We term this “remote knock-on”, which was first proposed from a simple model ion channel for the enhancement of ion permeability^41^. This unique remote knock-on and one-at-a-time permeation pattern observed in our MD simulations occurs due to both structural features of RyR and strong repulsive interactions of divalent cations, which was not observed in previously studied ion channels.

Another distinct permeation feature of RyR is that Ca^2+^ ions are nearly fully hydrated during the translocation along the narrow pore region. Previous studies have shown that K^+^ is nearly fully dehydrated^11,16^ and Na^+^ is partially dehydrated^13,14^ during permeation, meaning all of the water molecules or at least several water molecules within the first solvation shell of the ions must be removed or replaced by other residues at the narrowest constriction sites. In fact, this dehydration process was believed to be the key factor responsible for the ion selectivity of the channels^13,17,42^, as different ions have different sized solvation shell and selectivity filters with different steric and chemical features can discriminate them by the free energy difference during the dehydration process. Interestingly, we did not observe such a dehydration behavior when Ca^2+^ was permeating through the RyR1 channel. As shown in Fig. 4, the number of water molecules are nearly constant in the narrow pore region and the oxygen atoms coordinating with the Ca^2+^ from the protein is nearly zero throughout, indicating that the first solvation shell of the Ca^2+^ is intact during permeation, and no dehydration occurred when the ion passing through the narrow constriction sites in the selectivity filter and lower gate. This finding provides a clear picture on the exact hydration states of Ca^2+^ ions as they pass through the pore, which is consistent with earlier speculation that ion dehydration may not be a significant component of selectivity or permeation^43^. From the structural point of view, the open-state RyR1 is wider than K^+^ and Na^+^ channels in the selectivity filter region (Fig. 4), and therefore sterically allows the Ca^2+^ to permeate with its first solvation shell intact. On the one hand, this makes the free energy barrier of Ca^2+^ translocation lower, allowing highly efficient Ca^2+^ permeation to regulate ion concentration in the cytosol for muscle contraction and heartbeat, which is otherwise hard to imagine as Ca^2+^ has a much higher dehydration energy compared to K^+^ and Na^+^. On the other hand, this probably also leads to the weaker ion selectivity of RyR1, compared to the highly selective K_V_, Na_V_, and Ca_V_ channels, since the powerful dehydration-based selectivity mechanism cannot be utilized here.

It should be noted that, we adopted a truncated RyR1 in our simulations. Although this covers the whole transmembrane region, it does not represent the entire protein. The large cytoplasmic domain, which is important for the gating and regulation of the RyR permeation, was not considered in the present study. Interestingly, Heinz et al.’s recent study of a less truncated RyR1 showed very similar binding sites to ours, although the specific PMF profile was not identical due to the different ion models and truncation sites adopted^30^. Therefore, we think the truncation may affect the ion motion after entering the cytoplasmic side, but the permeation behavior through the transmembrane pore and the binding sites along this path should remain valid. We also calculated the conductance of the pore using the fluctuation-dissipation theorem by monitoring the spontaneous Ca^2+^ permeation events and obtained a value of ~139 pS (Supplementary Fig. 6), which is in very good agreement with the value calculated under a transmembrane potential mentioned above (~141 pS), indicating nice convergence of the simulations.

**Fig. 7:**
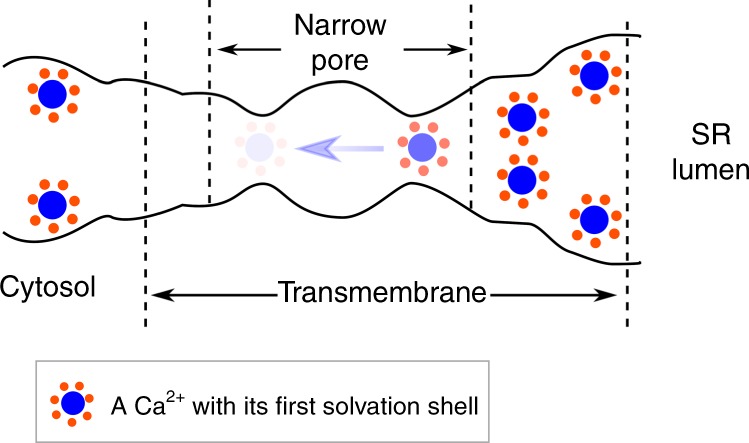
Model for the Ca^2+^ permeation mechanism of RyR1. There is only one Ca^2+^ in the narrow pore region, with a longer residence time at the selectivity filter constriction site (darker color), and a shorter residence time at the gate constriction (lighter color).

In summary, we developed a multi-site Ca^2+^ model which behaves well when studying its interactions with proteins. It is entirely consistent with the widely used CHARMM force field, and we are expanding to other force fields and divalent ions as well. With the new Ca^2+^ model, we revealed the detailed Ca^2+^ permeation process through the open-state RyR1, and discovered that multiple Ca^2+^ ions can accumulate in the wide upper selectivity filter, and then permeate through the narrow pore region following a remote knock-on and one-at-a-time pattern, in which the permeating Ca^2+^ is fully hydrated with an intact first solvation shell (Fig. 7), distinct from the widely studied K^+^ and Na^+^ channels. The transmembrane pore region is wide enough to allow hydrated cations to permeate and therefore facilitate a large ion conductance. The selectivity filter of RyR1 is shorter and wider than the well-studied K_V_ and Na_V_ channels and cannot discriminate cations by their solvation shells, leading to a weak selectivity among cations by the so-called charge-space competition mechanism. Our simulation results shed new light on the high efficiency and weak cation selectivity of the Ca^2+^ permeation through the RyR channels.

## Methods

### Simulations for parameterization

All the MD simulations for the ion model parameterization were performed with OpenMM (version 7.0.1)^44^, as the package is highly flexible and can be easily customized with a Python interface, and the CHARMM force field^45^ (version C36_Aug15) was utilized in this work.

We built an ion-in-water system to optimize the ion properties in the water, which consists of one multi-site Ca^2+^ ion in a cubic water box of 3 × 3 × 3 nm^3^. The TIPS3P water model was used in consistency with the CHARMM force field. In our simulations, NPT ensembles were generated by integrating the Langevin dynamics with a time-step of 2 fs and a collision frequency of 5 ps^−1^. The temperature was maintained at 298 K, and the pressure was regulated at 1 bar using a Monte Carlo barostat. Water molecules were kept rigid during simulations, and the cutoff of non-bonded interactions was 1 nm. For calculations of the radial distribution function, coordination number, and residence time, a 20-ns trajectory was generated. For hydration free energy calculations, 1-ns trajectories were generated for each of the 14 alchemical states (please also see below). The hydration free energy was estimated using the python implementation of the multistate Bennett acceptance ratio downloaded from https://github.com/choderalab/pymbar. The properties related to the radial distribution function were calculated using MDTRAJ^46^.

To optimize the ion-protein interactions, we used the Ca^2+^-Protein systems previously investigated by Li et al.^9^, and used OpenMM and CHARMM force field to perform the calculations.

### Fitness functions for optimization

We define two fitness functions to optimize the parameters of our multi-site Ca^2+^ model. One is designated as the protein-fitness function $(\lambda _{\mathrm{p}}^2)$ and the other as the water-fitness function $(\lambda _{\mathrm{w}}^2)$. The protein-fitness function is defined against a dataset of quantum-mechanically calculated Ca^2+^-protein binding energies, $\Delta E_{p,f}^{{\mathrm{QM}}}$, where *p* identifies the index among *N*_p_ (=10) proteins, and *f* indexes *N*_f_ (=21) trajectory snapshots for each protein. The formula for $\lambda _{\mathrm{p}}^2$ is

1 $$\lambda _{\mathrm{p}}^2 = \mathop {\sum}\limits_{p = 1}^{N_{\mathrm{p}}} {\left[ {\overline {\Delta E} _p^{{\mathrm{MM}}} - \left( {\overline {\Delta E} _p^{{\mathrm{QM}}} + \Delta E_{\mathrm{C}}} \right)} \right]^2} ,$$

where $\overline {\Delta E} _p^{\mathrm{X}} = \mathop {\sum}\nolimits_f {\Delta E_{p,f}^{\mathrm{X}}/N_{\mathrm{f}}\, ({\mathrm{X}} = {\mathrm{QM}},{\mathrm{MM}})}$, and MM indicates the molecule-mechanical results. Due to limitations of the methodology level and the basis-set size used in quantum-mechanical calculations, a systematic correction of binding energies (**Δ***E*_C_ = 10 kcal/mol) is added to $\overline {\Delta E} _p^{{\mathrm{QM}}}$ following Li et al.’s strategy^9^.

In addition to Ca^2+^-protein interactions, we also optimize our model to reproduce its energetical, structural, and dynamic properties in water. Specifically, these properties include the hydration free energy (**Δ***G*_h_), the first-peak position of radial distribution function (*R*_1_), the coordination number (*N*_C_), and the residence time of water in the first coordination shell (*τ*_R_). The water-fitness function is defined as

2 $$\lambda _{\mathrm{w}}^2 = \left( {\frac{{\Delta G_{\mathrm{h}}^t - \Delta G_{\mathrm{h}}^e}}{{\Delta G_{\mathrm{h}}^w}}} \right)^2 \,+\, \left( {\frac{{R_1^t - R_1^e}}{{R_1^w}}} \right)^2 \,+\, \left( {\frac{{N_{\mathrm{C}}^t - N_{\mathrm{C}}^e}}{{N_{\mathrm{C}}^w}}} \right)^2 \,+\, \left( {\frac{{\tau _{\mathrm{R}}^t - \tau _{\mathrm{R}}^e}}{{\tau _{\mathrm{R}}^w}}} \right)^2,$$

where quantities with superscripts of *t*, *e*, and *w* stand for theoretical, experimental, and weighting values, respectively.

### Target properties of Ca^2+^ in water

We followed the approach of Mamatkulov et al.^47^ to determine the target hydration energy of Ca^2+^ (−1504 kJ/mol) as

3 $$\Delta G_{\mathrm{h}}^{\mathrm{e}}\left( {{\mathrm{Ca}}^{2 + }} \right) = \Delta G\left( {{\mathrm{CaCl}}_2} \right) - 2\Delta G_{\mathrm{h}}\left( {{\mathrm{Cl}}^ - } \right) - \Delta G_{{\mathrm{press}}} - \Delta G_{{\mathrm{surf}}},$$

where **Δ***G*(CaCl_2_) is the measured hydration energy of CaCl_2_^48^, Δ*G*_h_(Cl^−^) the theoretical hydration energy determined from Smith-Dang parameters^49^ for Cl^−^, Δ*G*_press_ the energy needed to compress one mole of ion gas at 1 atm into a liter, and Δ*G*_surf_ the energy change of crossing the air-water interface for 1 mol of ions. Marcus assessed the Ca–O internuclear distances in calcium salt solutions from different studies and concluded that the generally consistent result is 0.242 nm^50^, which was used as our target value for $R_1^{\mathrm{e}}$. A recent neutron diffraction study^51^ revealed that the average number of water molecules in the first hydration shell of Ca^2+^ is close to 7, which we took as the target value for *N*_C_^e^. It is difficult to experimentally determine the residence time of water molecules in the first hydration shell of the calcium ion^52^, and a nuclear magnetic resonance (NMR) study estimated its value to be <100 ps^27^. Since the existing Ca^2+^ models generally overestimate *τ*_R_, we set $\tau _{\mathrm{R}}^{\mathrm{e}}$ as zero in the course of optimization and checked the final result so as to be the same order of magnitude as 100 ps. The numerical values for the targeted experimental properties and their corresponding weights are listed in Supplementary Table 2.

### Target binding energies of Ca^2+^ with proteins

We followed Li et al.’s^9^ protocol to calculate the binding energies between Ca^2+^ and proteins. As noted by Li et al., the default single-site model of Ca^2+^ in the CHARMM C36 force field generally overestimates the Ca^2+^-protein binding energies by 150–200 kcal/mol with respect to quantum-mechanical binding energies. They selected 10 high-resolution crystal structures of Ca^2+^-binding enzymatic proteins (PDB IDs shown in Fig. 1) and performed MD simulations of the solvated proteins. From the equilibrated trajectories, 21 conformation snapshots were extracted for each protein and truncated to include atoms within a sphere of ~0.55 nm around the ion. Quantum mechanical calculations were then carried out with the truncated models to obtain a dataset of binding energies, which is used in this work to optimize the multi-site Ca^2+^ model. For the systematic correction of binding energies (**Δ***E*_C_) that is added to $\overline {\Delta E} _p^{{\mathrm{QM}}}$, we use an estimation of 10 kcal/mol as Li et al. did in their work. All the ab initio and Drude binding energies were from the original work by Li et al.^9^.

### Calculation of properties from simulations

The theoretical hydration energy, $\Delta G_{\mathrm{h}}^{\mathrm{t}}$, consists of two terms, i.e.

4 $$\Delta G_{\mathrm{h}}^{\mathrm{t}} = \Delta G_{\mathrm{h}}^{{\mathrm{alchem}}} + \Delta G_{\mathrm{h}}^{{\mathrm{fs}}}.$$

The first term refers to the free energy change corresponding to alchemically switching off the ion-water electrostatic and LJ interactions, and the second term is a correction due to the finite-size simulation box. The alchemical transformation for turning off the electrostatic interaction was performed with 10 intermediate states, during which the charge of ion was sequentially reduced by a tenth of the total charge. After that, it took four uniform intermediate states with a soft-core potential (*α* = 0.5)^53^ to annihilate the LJ interaction. MD trajectories (1 ns) of one ion in a cubic water box (3 nm) were used to estimate the alchemical free energy by the method of multistate Bennett acceptance ratio^54^. For the finite-size correction, we took the formula derived by Hummer et al.^55^,

5 $$\Delta G_{\mathrm{h}}^{{\mathrm{fs}}} = {\mathrm{Z}}^2{\mathrm{e}}^2\left[ { - \frac{{\xi _{{\mathrm{EW}}}}}{{2\varepsilon _{\mathrm{r}}}} + \left( {1 - \frac{1}{{\varepsilon _{\mathrm{r}}}}} \right)\frac{{\pi R_1^{{\mathrm{t}^2}}}}{{3L^3}}} \right],$$

where Ze is the ion charge, *ξ*_EW_ is the Wigner potential, *ε*_r_ (=82) is the relative dielectric constant, and *L* (=3 nm) is the box size.

The first-peak position of the radial distribution function $R_1^{\mathrm{t}}$ was calculated using the MDTRAJ software^46^ from a 20-ns trajectory, and the coordination number $N_{\mathrm{C}}^{\mathrm{t}}$ was computed by the integration of the first peak. We followed the definition described by Impey et al.^56^ to calculate the residence time $\tau _{\mathrm{R}}^{\mathrm{t}}$. First, the residence time distribution $n_{{\mathrm{ion}}}(t)$ was computed as

6 $$n_{{\mathrm{ion}}}\left( t \right) = \frac{1}{{N_{\mathrm{t}}}}\mathop {\sum }\limits_{n = 1}^{N_{\mathrm{t}}} \mathop {\sum }\limits_{j = 1}^{N_{\mathrm{w}}} P_j\left( {t_n,t;t^ \ast } \right),$$

where *N*_t_ is the number of time frames and *N*_w_ is the number of water molecules. *P*_*j*_(*t*_*n*_, *t*; t^*^) takes a value 1 if the water molecule *j* stays in the first hydration shell from *t*_*n*_ to *t*_*n*_ + *t* without leaving for any period larger than t^*^, and takes the value 0 otherwise. Then, *n*_ion_(*t*) was fitted with $n_0\exp ( - t/\tau _{\mathrm{R}}^{\mathrm{t}})$ to obtain $\tau _{\mathrm{R}}^{\mathrm{t}}$.

The molecule-mechanical Ca^2+^-protein binding energies $(\Delta E_{p,f}^{{\mathrm{MM}}})$ were calculated as the potential energy difference between Ca^2+^-bound and Ca^2+^-free configurations. Since our model of Ca^2+^ has a geometry of octahedron, the total energy of a Ca^2+^-bound system depends on the orientation of the octahedron. Thus, we first minimized the energy with respect to the orientation of the octahedron and obtained *E*_min_(Ca^2+^ + Protein), and then calculated the Ca^2+^-protein binding energy using the formula **Δ***E*^MM^ = *E*_min_(Ca^2+^ + Protein) − *E*(Ca^2+^) − *E*(Protein).

### Optimization strategy

First, we did a thorough scan and prescreening, using the conventional optimization routines (such as conjugate gradient and basin hopping), random sampling, and sifting the parameter space by the properties described above in sequence. Initially, we focused on six parameters (*b*_CD_, *Q*_C_, *ε*_C_, *σ*_C_, *ε*_D_, *σ*_D_), i.e., treating interactions with water and protein on an equal footing. The searched parameter space as shown in Supplementary Table 3 is determined from previous works^25,26^, and we substantially extended the range of *Q*_C_, *ε*_C_, and *ε*_D_ based on our optimization results. However, after systematic optimization, the calculated water properties and protein binding energies were still slightly off from the target values. Therefore, we further split (*ε*_C_, *σ*_C_) into ($\varepsilon _{\mathrm{C}}^{{\mathrm{NW}}}$, $\sigma _{\mathrm{C}}^{{\mathrm{NW}}}$) and ($\varepsilon _{\mathrm{C}}^{\mathrm{W}}$, $\sigma _{\mathrm{C}}^{\mathrm{W}}$), thus calculating the interactions of Ca^2+^ with protein and water separately. In fact, we took a two-step strategy to optimize our multi-site model. First, the water-fitness function $\lambda _{\mathrm{w}}^2$ was minimized to obtain optimal values for (*b*_CD_, *Q*_C_, $\varepsilon _{\mathrm{C}}^{\mathrm{W}}$, $\sigma _{\mathrm{C}}^{\mathrm{W}}$, *ε*_D_, *σ*_D_). Second, with the parameters of (*b*_CD_, *Q*_C_, *ε*_D_, *σ*_D_) fixed, the protein-fitness function $\lambda _{\mathrm{p}}^2$ was scanned with a two-dimension grid to find the best values of ($\varepsilon _{\mathrm{C}}^{{\mathrm{NW}}}$, $\sigma _{\mathrm{C}}^{{\mathrm{NW}}}$) that reproduce quantum-mechanical Ca^2+^-protein binding energies. Due to the roughness of $\lambda _{\mathrm{w}}^2$ owing to the stochastic nature of the calculation, we developed a simple yet effective steep-descent-like algorithm that alternatingly searches along each dimension of the parameter space instead of the gradient. The parameter searching along each dimension was performed at linearly spaced steps, which were then finely tuned near the local minimum of a finite interval centering at the current minimal point. We used this strategy to further optimize the top parameters obtained in the initial stage of searching, which indeed yielded more satisfactory results. We found that the minimization mainly took place in the dimensions other than $\varepsilon _{\mathrm{C}}^{\mathrm{W}}$ and *ε*_D_, which indicated that the landscape of the water-fitness function is much smoother along these two dimensions. Therefore, we selected a couple of combinations of ($\varepsilon _{\mathrm{C}}^{\mathrm{W}}$, *ε*_D_) based on the scanning results and carried out further optimization until the fitness value was sufficiently low. The final optimized parameters are presented in Supplementary Table 4.

### Details of permeation simulations

To study the permeation of Ca^2+^ through RyR1, we performed MD simulations with GROMACS^57^ version 5.1.3 with the CHARMM36 force filed^45^ and the TIPS3P water model. The channel pore domain (residue 4820–4956) of the cryo-EM structure of the open-state RyR1^21^ (PDB ID: 5TAL [10.2210/pdb5TAL/pdb], resolution: 4.3 Å) was used as the starting structure. The resolution is not very high, but should be good enough for the present study as the open-state pore of RyR1 is relatively wide and the subtle configurations within the pore may affect the ion permeation to a minor extent, and the side chains are free to move in our MD simulations to sample low-energy local conformations. The OPM^58^ web server and Membrane Builder in CHARMM-GUI^59^ were used to build four POPC-RyR1 simulation systems.

A POPC-RyR1 simulation system with ~150 mM of calcium ions and a POPC-RyR1 simulation system with ~250 mM of potassium ions were built to calculate the conductance of the calcium ions and potassium ions with a transmembrane potential of 100 mV. The ion concentrations were chosen to match the experimental conditions under which the conductance was measured^31^. As K^+^ exhibits a higher saturation concentration than Ca^2+^, we used a [Ca^2+^] of 150 mM and [K^+^] of 250 mM in our simulations. Six 500-ns trajectories were simulated for the former system, and three 300-ns trajectories for the latter. By counting the number of permeation events (*N*_p_) for a trajectory of certain time length (*t*_traj_) under a transmembrane potential of *V*_tm_, the ion conductance *C*_ion_ was calculated with:

7 $$C_{{\mathrm{ion}}} = I/V_{{\mathrm{tm}}} = \frac{{N_{\mathrm{p}} \times Q_{{\mathrm{ion}}}}}{{t_{{\mathrm{traj}}}}}/V_{{\mathrm{tm}}}$$

where *Q*_ion_ is the charge of the permeating ion.

Another POPC-RyR1 simulation system with ~150 mM calcium ions was built to calculate the conductance of the calcium ions with our new calcium model with the same transmembrane potential of 100 mV. As the ion model cannot be simulated as a rigid body with GROMACS, we used bond and angle restraints to make the multi-site Ca^2+^ model as rigid as possible, which affect the thermodynamics of the Ca^2+^ to a negligible extent (please refer to Supplementary Table 5). Six independent 500-ns trajectories were conducted. And, one 800-ns trajectory was run for the same system but without an electric field to obtain the stable binding sites of the calcium ions in the open-state RyR1, which would be more comparable to ion positions in the cryo-EM density maps than with an electric field.

Based on the 800-ns simulation trajectory, we estimated the free energy profile of Ca^2+^ along the pore. We first calculated the Ca^2+^ density in the simulation system with a grid-spacing of 1 Å, and then we determined the maximum density of Ca^2+^ at a certain position along the axis of the pore: $\rho ^ \ast \left( z \right) = {{\max }_{x,y} }\rho (x,y,z)$. Then the free energy profile is calculated as $G\left( z \right) = - RT\ {\mathrm{ln}}\ \rho ^ \ast \left( z \right)$.

All the simulation systems were first equilibrated with the standard CHARMM-GUI equilibration protocol followed by the production simulations with the harmonic position restraints applied on the *α* carbon of the protein (with a force constant of 1000 kJ mol^−1^ nm^−2^). The position restraints were applied to make sure the open-state conformation was stable in the absence of the large intracellular domain and the caffeine and ATP bound to it, but the atoms other than *α* carbon, including all the side chains of the transmembrane domain, were fully flexible in our simulations, allowing a certain degree of flexibility of the protein (Supplementary Fig. 7). For all the production simulations, the periodic boundary conditions were used and the time step was 2 fs. The v-rescale algorithm with a time constant of 0.5 ps was used to maintain the temperature at 310 K, and the Parrinello-Rahman algorithm^60^ with a time constant of 1 ps was used to maintain the pressure at 1.0 bar. The Particle-mesh Ewald method^61^ was used to calculate electrostatics, and the cut-off length of the van der Waals interaction was 1.2 nm.

### Reporting summary

Further information on research design is available in the Nature Research Reporting Summary linked to this article.

## Supplementary information

Supplementary Information

Reporting Summary

Description of Additional Supplementary Files

Supplementary Movie 1

Supplementary Movie 2

Supplementary Movie 3

## Data Availability

The data that support the findings of this study are available from the corresponding author upon reasonable request. The source data underlying Figs. 1, 2b, c, 3a, and 4–6 and Supplementary Figs. 1, 2, 3c, d, 4b, c, and 5–7 are provided as a Source Data file.

## Source data

Source Data
