# Supplementary material for: The Ca2+ permeation mechanism of the ryanodine receptor revealed by a multi-site ion model
Source: Nat Commun. 2020 Feb 17;11:922. doi: 10.1038/s41467-020-14573-w (PMC7026163; doi:10.1038/s41467-020-14573-w)
Supplement: Supplementary file 1 — Supplementary Information [file 41467_2020_14573_MOESM1_ESM.pdf]

Supplementary Information

The Ca^{2+} Permeation Mechanism of the Ryanodine Receptor Revealed by a Multi-Site Ion Model

Zhang et al.

Supplementary Methods

1. *Ab initio* Calculation of Ca^{2+}-Protein Binding Energies

The *ab initio* Ca^{2+}-protein binding energies were from the work of Li et al.[1]. The truncated Ca^{2+}-protein structures are available for download from the original paper[1]. The B3LYP functional with the CEP-121 basis set was used for the calculations[2–6], with the basis set superposition error (BSSE) corrections for quantum mechanics (QM) calculations[7]. This setting was chosen to balance the accuracy and computational cost. By comparing with calculations using a higher level theory (MP2) for small benchmark systems, a systematic correction of 10 kcal/mol was added to obtain the final binding-energy dataset, which was used for our model optimization and designated as QM+10 in the main text. Please refer to Li et al.'s original work for more details[1].

2. Born-Oppenheimer Molecular Dynamics

We performed DFT-BOMD to estimate the residence time of water molecules in the first solvation shell of Ca^{2+} using VASP[8]. The initial configuration of the system was taken from an equilibrium trajectory simulated using classical MD with the CHARMM force field[9]. The system consisted of one Ca^{2+} cation and 64 water molecules in a cubic box with edge length of 1.25 nm. A uniform counter-charge background was implicitly assumed. A plane-wave basis set with 400 eV cutoff was used to expand the wave function of electrons. The core-valence interaction was represented by PAW potentials[10]. The exchange-correlation functional was approximated by LDA or GGA-PBE. Only Γ-point was used for the k-space integration. The system's temperature was regulated at 300 K using a Nosé-Hoover thermostat[11,12] and trajectories were sampled in a NVT ensemble with a time step of 1 fs. The Ca^{2+}-O distance variations as a function time are presented in Supplementary Figure 2.

3. PMF Calculation of Ca^{2+} Dissociating from Calpain

We chose a Ca^{2+}-bound calpain structure (PDB code: 1ALV), which was not in the fitting dataset of proteins, to validate our model by calculating the Ca^{2+}-protein binding affinity. The reported experimental binding affinity was 8.3 kcal/mol[13].

The protein was solvated in a water box of 8.6×8.6×9.0 nm^3 with 0.1 M $CaCl_2$. The backbone of the protein was position-restrained using a harmonic potential with a force constant of 400 $kJ \cdot mol^{-1} \cdot nm^{-2}$. The reaction coordinate of dissociation was defined as the vector between the Ca^{2+} and the center of mass of the binding loop consisting of the residues numbered from 180 to 188. A flat-bottom restraint potential (http://manual.gromacs.org/documentation/) with a force constant of 1000 $kJ \cdot mol^{-1} \cdot nm^{-2}$ was applied to confine the dissociating Ca^{2+} in a cylinder with a radius of 0.6 nm. The potential of mean force (PMF) was calculated with the umbrella sampling method by pulling the Ca^{2+} along the reaction coordinate from the equilibrium binding site to the bulk water where the PMF curve levels off. To get convergence, the reaction coordinate was sampled with 47 windows for the CHARMM Ca^{2+}, and 35 windows for our model. The window size was originally set to be 0.5 Å, and additional windows had to be added to obtain converged PMFs for both cases. For each window, a 40-ns trajectory was simulated for sampling after the system was equilibrated for 1 ns. All the simulation were carried out under the NPT ensemble at 300 K and 1.0 bar. All the other parameters were the same as in the MD method in the main text. Finally, PMF profiles were calculated using the weighted histogram analysis method as implemented in the software of GROMACS[14,15]. The calculated PMF profiles are presented in Supplementary Figure 3.

4. Electrostatic Potential Profile along the RyR1 Pore Axis

The electrostatic potential across the channel was calculated by solving the Poisson's equation using the APBSmem program[16]. A dielectric constant of 2 was assigned to the protein as well as the region of membrane, and 78 to the solvent. The membrane region with a thickness of 38

Å was defined by the average z coordinates of P atoms in the upper and lower leaflets of the lipid bilayer, while a cylinder region with a radius of 20 Å was excluded for the protein. The calculations were performed in the presence of 150 mM KCl or $CaCl_2$, with or without an explicit ion locating near the gate constriction. Only the electrostatic potential profiles along the pore axis are shown in Supplementary Figure 4.

Supplementary Tables

Supplementary Table 1. Comparison of binding energies of Ca^{2+} with ten chosen proteins calculated by *ab initio* method (QM+10), Drude model (Drude), our model (CAM) and CHARMM force field (CHARMM). The first two data sets were taken from the work of Li et al.[1]. The unit is kcal/mol.

PDB ID	QM+10	Drude	Diff.	CAM	Diff.	CHARMM	Diff.
1A4V	-796	-794	2	-799	-3	-966	-170
1BLI	-619	-689	-70	-627	-8	-799	-180
1EXR	-724	-712	12	-763	-39	-934	-210
1RWY	-619	-593	26	-636	-17	-826	-207
2AAA	-963	-937	26	-966	-3	-1126	-163
2UUY	-776	-752	24	-722	54	-877	-101
3ICB	-699	-676	23	-680	19	-788	-89
3LI3	-728	-725	3	-740	-12	-857	-129
3TZ1	-808	-789	19	-810	-2	-963	-155
4KTS	-779	-778	1	-771	8	-932	-153
Average			6.6		-0.3		-155.7
SD			28.8		24.4		40.3

Supplementary Table 2. Target experimental properties, property weights and theoretical values calculated using the optimized parameters.

	experimental	weight	theoretical
ΔG_{h} (kJ/mol)	-1504.0	5.0	-1503.9
R_1 (nm)	0.242	0.002	0.2422
N_{C}	7.0	0.25	6.99
τ_{R} (ps)	< 100	50	75

Supplementary Table 3. The parameter space scanned for the optimization of the multi-site Ca^{2+} model.

b_{CD} (nm)	Q_{C} (e)	ε_{C} (kJ/mol)	σ_{C} (nm)	ε_{D} (kJ/mol)	σ_{D} (nm)
[0.05, 0.15]	[-8, 2]	[0.1, 10.1]	[0.20, 0.32]	[0.1, 10.1]	< 0.01

Supplementary Table 4. The optimized parameters.

b_{CD} (nm)	Q_{C} (e)	$\varepsilon_{\mathrm{C}}^{\mathrm{W}}$ (kJ/mol)	$\sigma_{\mathrm{C}}^{\mathrm{W}}$ (nm)	$\varepsilon_{\mathrm{C}}^{\mathrm{NW}}$ (kJ/mol)	$\sigma_{\mathrm{C}}^{\mathrm{NW}}$ (nm)	ε_{D} (kJ/mol)	σ_{D} (nm)
0.0905	-3.69	3.90	0.2418	6.78	0.2670	2.2	0.0095

Supplementary Table 5. The rigid and flexible multi-site Ca^{2+} models with the same non-bonded parameters show negligible differences in the ion properties in water.

	$\Delta G_{\mathrm{h}}^{\mathrm{e}}$ (kJ/mol)	R_{1}^{e} (nm)	$N_{\mathrm{C}}^{\mathrm{e}}$	$\tau_{\mathrm{R}}^{\mathrm{e}}$ (ps)
Flexible	-1503.9	0.2422	6.99	75
Rigid	-1503.3	0.2424	6.94	96

Supplementary Figures

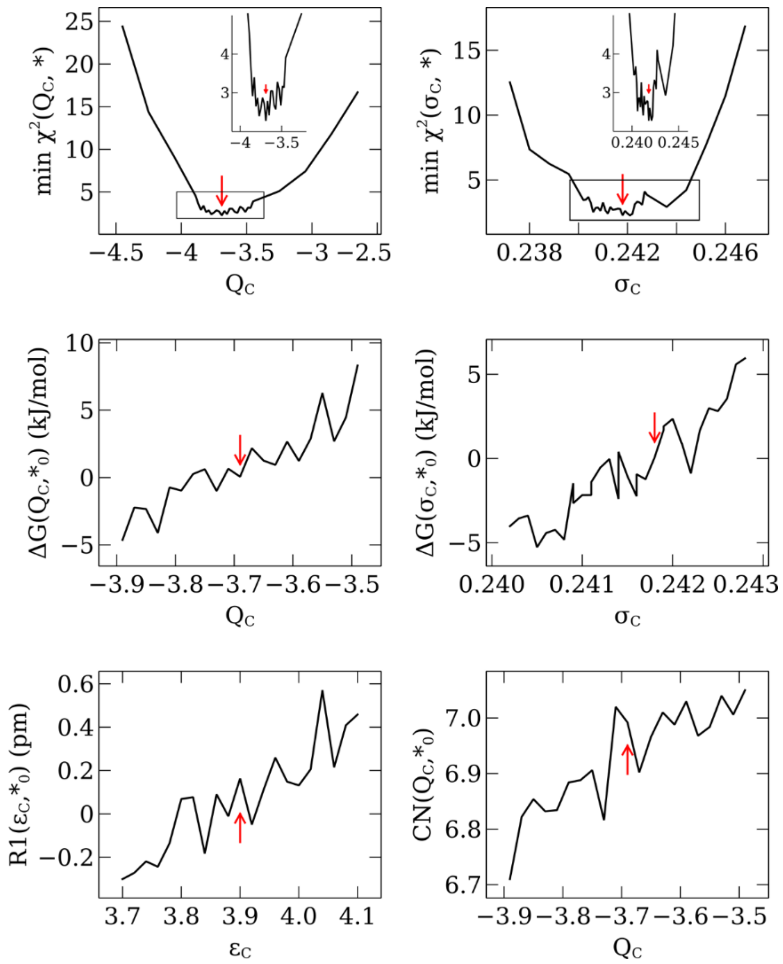

Supplementary Figure 1. Convergence of parameter optimization, showing variation of fitness and property values as functions of parameters in a local searching that leads to the chosen set of parameters. The insets in the upper two panels show the rescaled region in the rectangular box. The positions of the chosen parameters are indicated by red arrows. min $\chi^2(Q_{\mathrm{C}},*)$ means the minimized fitness value with the central charge being fixed as Q_{C}. $\Delta G(Q_{\mathrm{C}},*_0)$ is the hydration energy calculated with the central charge being Q_{C} and other parameters fixed at the optimal values. Other designations can be similarly interpreted. For ΔG and $R1$, the optimization targets have been set to 0 by subtracting the experimental values. Source data are provided as a Source Data file.

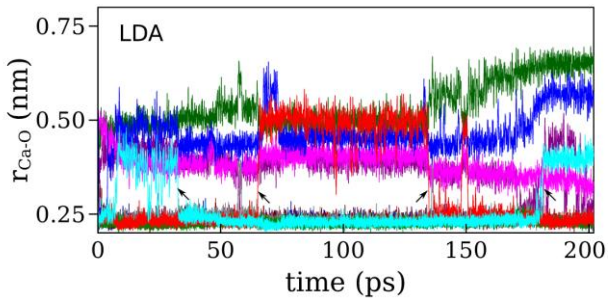

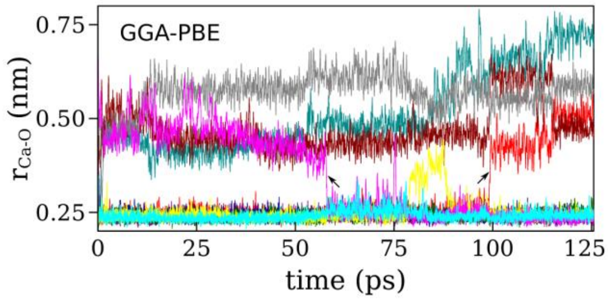

Supplementary Figure 2. Variation of distance between Ca^{2+} and water oxygen as a function of time. Only those water molecules that enter or leave the first shell are shown. The non-transient events of water exchanging between the first shell and bulk are indicated by arrows. Source data are provided as a Source Data file.

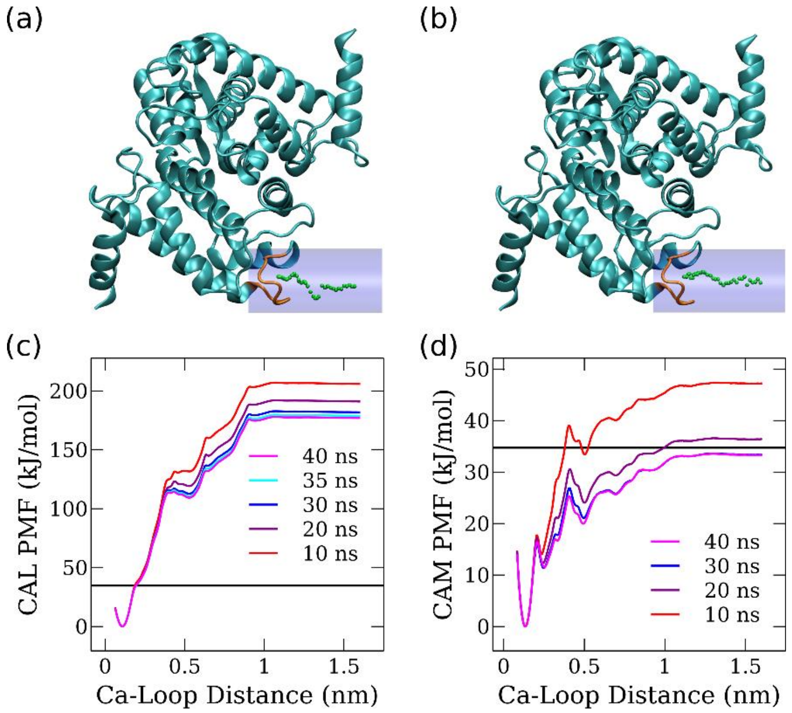

Supplementary Figure 3. The calpain structure (a, b) and the PMF profiles (c, d) of a Ca^{2+} dissociating from its binding site in the calpain protein (PDB ID: 1ALV), calculated with the CHARMM (a, c) or our Ca^{2+} model (b, d). The cylinder regions with a flat-bottom restraint potential are shown with transparent blue rectangles, and the binding loop is shown in orange in (a) and (b). The average positions of Ca^{2+} in all the sampling windows are shown as green spheres to indicate the dissociation pathway. The convergence of PMF is shown with multiple profiles generated with trajectories of different lengths for each sampling window. The black horizontal line corresponds to the experimental binding affinity (34.7 kJ/mol). The calculated binding free energies with the default Ca^{2+} (CAL) and our Ca^{2+} model (CAM) are 168.4 kJ/mol and 24.7 kJ/mol, respectively. The calculation was carried out following Woo and Roux's work[17]. The binding constant was first determined by integrating PMF with $K_{\mathrm{eq}} = S_{\mathrm{cylinder}} \int_{\mathrm{site}} \mathrm{d}z \; e^{-\beta[w(z)-w(z^*)]}$ and then the binding affinity was calculated by $\Delta G_{\mathrm{bind}} = k_{\mathrm{B}} T \ln(K_{\mathrm{eq}} C^{\circ})$ with $C^{\circ} = 1 \text{ mol/liter}$. Source data of (c) and (d) are provided as a Source Data file.

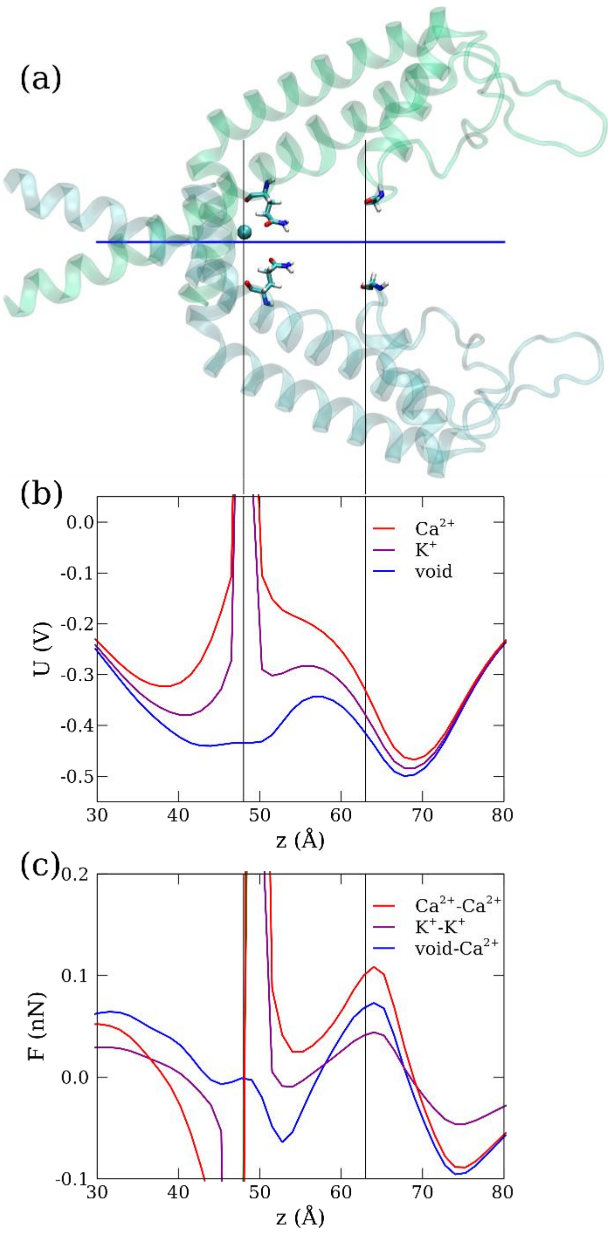

Supplementary Figure 4. The pore structure of RyR1 (a), the electrostatic potential profiles (b) and the repulsive forces experienced by a Ca^{2+} or K^{+} (c) along the pore axis of RyR1, when there is one Ca^{2+} (red line), one K^{+} (purple line) or no explicit ion (blue line) near the gate. The blue line in (a) indicates the axis of the pore and the cyan sphere indicates the ion location near the gate. The Ca^{2+} near the gate will exert an additional repulsive force of ~32 pN on the Ca^{2+} at the selectivity filter (SF). The forces experienced by the SF ions are 100 pN, 68 pN and 41 pN for the Ca^{2+}-Ca^{2+}, void-Ca^{2+} and K^{+}-K^{+} (gate occupant – SF occupant) situations, respectively. Source data of (b) and (c) are provided as a Source Data file.

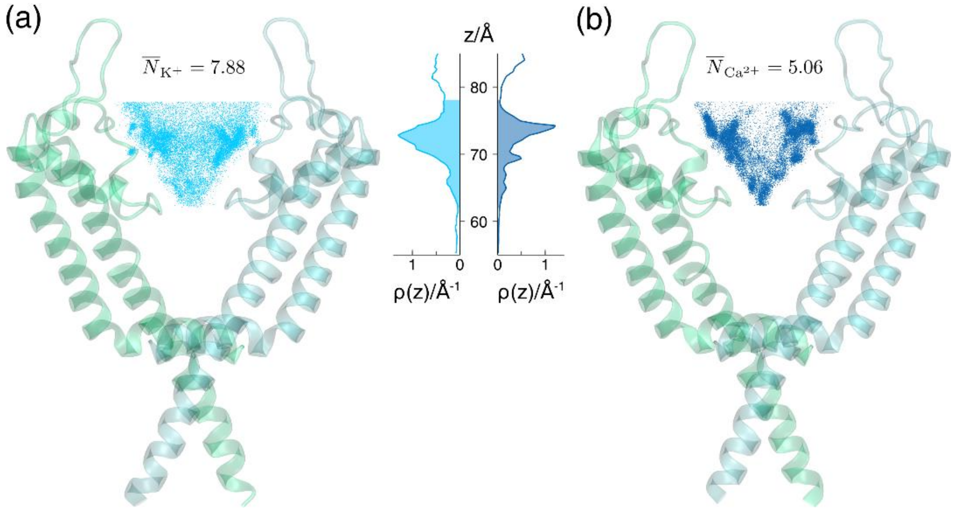

Supplementary Figure 5. The average number of ions occupying the selectivity filter of RyR1. The number was calculated for K^+ (a) or Ca^{2+} (b) in the presence of a 100-mv transmembrane potential. The ions located above the selectivity filter constriction site and within the pore were considered. The positions of the ions, represented by dots in the protein structures, were sampled from 6 × 500-ns trajectories (Ca^{2+}) and 3 × 300-ns trajectories (K^+). The ion number densities along the channel axis were given in the middle panel, and the integration of the shaded area yielded the average number of ions within the filter. In this figure, K^+ and Ca^{2+} are referred to by the cyan and blue colors, respectively. Source data are provided as a Source Data file.

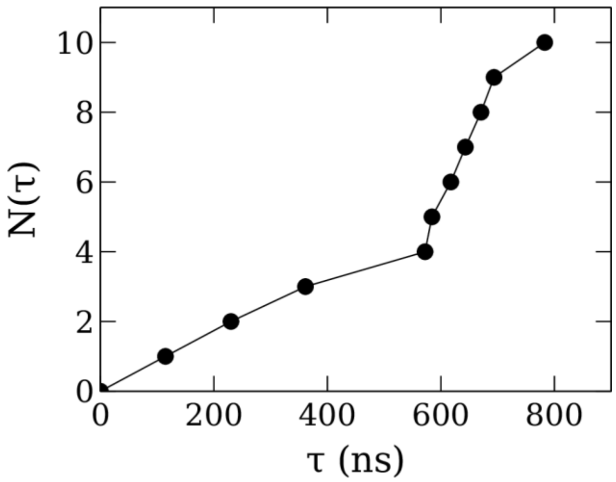

Supplementary Figure 6. The cumulative number of spontaneous permeation events as a function of time in an equilibrium trajectory for a system consisting of Ca^{2+} (our model) and RyR1. Based on this, the conductance calculated according to the fluctuation-dissipation theorem was 139 pS (Eq. 102 in Roux et al.'s review paper[18]), which agrees well with the conductance (141 pS) calculated under a transmembrane potential of 100 mV. Source data are provided as a Source Data file.

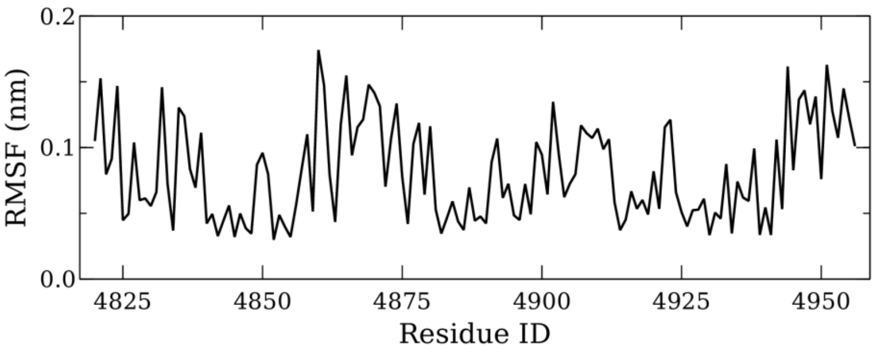

Supplementary Figure 7. The root-mean-square fluctuation (RMSF) of the RyR1 residues in our MD simulations, calculated for the heavy atoms and averaged for the four chains in the $6 \times 500\ ns$ trajectories. Source data are provided as a Source Data file.

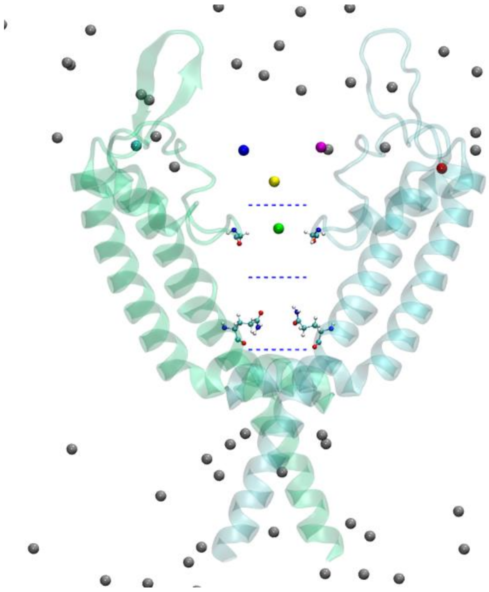

Supplementary Figure 8. Description of the permeation movies. The first frame of a typical permeation trajectory (please also see the SI movie1). The same trajectory was used for making Fig. 5a. Only two of the four chains are depicted for clarity. The calcium ions that permeate through the channel are colored as in Fig. 5a, and the remaining ions are in gray. The channel is divided into two chambers containing the binding sites at the selectivity filter (SF) and the gate (GT) by the dashed lines. The bottleneck residues (GLY-4894 at SF and GLN-4933 at GT) are drawn in the ball-and-stick style. Similar movies were made for the simulations with the default Ca^{2+} and K^{+} models in CHARMM (SI movies 2&3). All biomolecule graphics were generated with VMD[19].

Supplementary References

1. Li, H. *et al.* Representation of Ion-Protein Interactions Using the Drude Polarizable Force-Field. *J. Phys. Chem. B* **119**, 9401–9416 (2015).
2. Becke, A. D. A new mixing of Hartree–Fock and local density-functional theories. *J. Chem. Phys.* **98**, 1372–1377 (1993).
3. Lee, C., Yang, W. & Parr, R. G. Development of the Colle-Salvetti correlation-energy formula into a functional of the electron density. *Phys. Rev. B* **37**, 785 (1988).
4. Stevens, W. J., Basch, H. & Krauss, M. Compact effective potentials and efficient shared-exponent basis sets for the first-and second-row atoms. *J. Chem. Phys.* **81**, 6026–6033 (1984).
5. Stevens, W. J., Krauss, M., Basch, H. & Jasien, P. G. Relativistic compact effective potentials and efficient, shared-exponent basis sets for the third-, fourth-, and fifth-row atoms. *Can. J. Chem.* **70**, 612–630 (1992).
6. Cundari, T. R. & Stevens, W. J. Effective core potential methods for the lanthanides. *J. Chem. Phys.* **98**, 5555–5565 (1993).
7. Xantheas, S. S. On the importance of the fragment relaxation energy terms in the estimation of the basis set superposition error correction to the intermolecular interaction energy. *J. Chem. Phys.* **104**, 8821–8824 (1996).
8. Kresse, G. & Furthmüller, J. Efficient iterative schemes for ab initio total-energy calculations using a plane-wave basis set. *Phys. Rev. B* **54**, 11169–11186 (1996).
9. Huang, J. *et al.* CHARMM36m: an improved force field for folded and intrinsically disordered proteins. *Nat. Methods* **14**, 71 (2017).
10. Kresse, G. & Joubert, D. From ultrasoft pseudopotentials to the projector augmented-wave method. *Phys. Rev. B* **59**, 1758–1775 (1999).
11. Nosé, S. A unified formulation of the constant temperature molecular dynamics methods. *J. Chem. Phys.* **81**, 511–519 (1984).

12. Hoover, W. G. Canonical dynamics: Equilibrium phase-space distributions. *Phys. Rev. A* **31**, 1695–1697 (1985).

13. Schymkowitz, J. W. *et al.* Prediction of water and metal binding sites and their affinities by using the Fold-X force field. *Proc. Natl. Acad. Sci.* **102**, 10147–10152 (2005).

14. Abraham, M. J. *et al.* GROMACS: High performance molecular simulations through multi-level parallelism from laptops to supercomputers. *SoftwareX* **1–2**, 19–25 (2015).

15. Hub, J. S., de Groot, B. L. & van der Spoel, D. g_wham—A Free Weighted Histogram Analysis Implementation Including Robust Error and Autocorrelation Estimates. *J. Chem. Theory Comput.* **6**, 3713–3720 (2010).

16. Callenberg, K. M. *et al.* APBSmem: a graphical interface for electrostatic calculations at the membrane. *PloS One* **5**, e12722 (2010).

17. Woo, H.-J. & Roux, B. Calculation of absolute protein–ligand binding free energy from computer simulations. *Proc. Natl. Acad. Sci. U. S. A.* **102**, 6825 (2005).

18. Roux, B., Allen, T., Bernèche, S. & Im, W. Theoretical and computational models of biological ion channels. *Q. Rev. Biophys.* **37**, 15–103 (2004).

19. Humphrey, W., Dalke, A. & Schulten, K. VMD – Visual Molecular Dynamics. *J. Mol. Graph.* **14**, 33–38 (1996).
