# Supplementary material for: The Ca2+ permeation mechanism of the ryanodine receptor revealed by a multi-site ion model
Source: Nat Commun. 2020 Feb 17;11:922. doi: 10.1038/s41467-020-14573-w (PMC7026163; doi:10.1038/s41467-020-14573-w)
Supplement: Supplementary file 3 — Description of Additional Supplementary Files [file 41467_2020_14573_MOESM3_ESM.pdf]

Description of Additional Supplementary Files

File Name: Supplementary Movie 1
Description: Ca^{2+} ions permeating through the open-state RyR1 in the molecular dynamics simulations with our new Ca^{2+} model. The permeating ions are highlighted with different colors other than grey.

File Name: Supplementary Movie 2
Description: No Ca^{2+} permeation through the open-state RyR1 observed in the molecular dynamics simulations with the default Ca^{2+} model.

File Name: Supplementary Movie 3
Description: K^+ ions permeating through the open-state RyR1 in the molecular dynamics simulations.
